# Maya Angelou Center for Health Equity Caregiver College (MC2): Focus Group Feedback from Tier 1 and Tier 3 Counties in North Carolina

**DOI:** 10.1002/alz.093560

**Published:** 2025-01-09

**Authors:** Allison M. Caban‐Holt, Doreen Rone Fortune, Ashley H. Sanderlin, Charlene Whitaker‐Brown, Ruby Tonnette Artis, Takiyah D. Starks, Shawnta Lloyd, Ashley Gregory, Goldie S. Byrd

**Affiliations:** ^1^ Maya Angelou Center for Health Equity, Wake Forest University School of Medicine, Winston‐Salem, NC USA; ^2^ North Carolina Agricultural & Technical State University, Greensboro, NC USA; ^3^ University of North Carolina, Charlotte, Charlotte, NC USA

## Abstract

**Background:**

Black/African American (AA) caregivers of patients with Alzheimer’s disease (AD) and related disorders can play a critical role in maintaining patient health. AD caregivers who face economic challenges may have less access to resources that may reduce caregiver burden they experience. The North Carolina Department of Commerce ranks the state’s 100 counties based on economic well‐being and assigns the most distressed counties as Tier 1, and least distressed as Tier 3. Focus groups were conducted to determine the needs of Black/AA AD caregivers in a Tier 1 and a Tier 3 county.

**Method:**

The Maya Angelou Center for Health Equity Caregiver College (MC2) is an educational program that aims to improve informal caregivers’ knowledge of AD. Focus groups were conducted with community stakeholders in Wilson (Tier 1) and Mecklenburg (Tier 3) counties in North Carolina. Focus group attendees were engaged in discussion on topics including a review of local resources to support caregiving, challenges that AD caregivers face, educational needs of caregivers and preferred format of an MC2 event.

**Result:**

Focus group participants (N = 15; Wilson = 8, Mecklenburg = 7) are African American (100%), with professional and community roles including clergy, caregivers, nursing, teaching, manager, community health worker, and local city officials. Findings suggest that significant needs in a Tier 1 county include lack of geriatric∖AD specialists, few caregiver support resources, and little qualified in‐home care assistance. In a Tier 3 county, structural barriers and physical access to resources were named as difficulties. Common issues for both counties included confusion by what insurance plans cover. The design for MC2 was requested as a 1‐day format in the Tier 3 County because of a large population of working caregivers, whereas in the Tier 1 County a multi‐day MC2 format was recommended due to the perception that people in the county would benefit more from a more intensive AD educational event.

**Conclusion:**

Needs in Tier 1 and Tier 3 counties differed, however, both groups need help navigating medical insurance. Results will be used to customize the MC2 events to meet the expressed needs of the stakeholders in each county.

